# High-precision geochronological constraints on the duration of ‘Dinosaur Pompeii’ and the Yixian Formation

**DOI:** 10.1093/nsr/nwab063

**Published:** 2021-04-12

**Authors:** Yuting Zhong, Magdalena H Huyskens, Qing-Zhu Yin, Yaqiong Wang, Qiang Ma, Yi-Gang Xu

**Affiliations:** State Key Laboratory of Isotope Geochemistry, Guangzhou Institute of Geochemistry, Chinese Academy of Sciences, China; CAS Center for Excellence in Deep Earth Science, China; Department of Earth and Planetary Sciences, University of California at Davis, USA; Department of Earth and Planetary Sciences, University of California at Davis, USA; State Key Laboratory of Palaeobiology and Stratigraphy, Nanjing Institute of Geology and Palaeontology and Center for Excellence in Life and Paleoenvironment, Chinese Academy of Sciences, China; State Key Laboratory of Isotope Geochemistry, Guangzhou Institute of Geochemistry, Chinese Academy of Sciences, China; State Key Laboratory of Geological Processes and Mineral Resources, School of Earth Sciences, China University of Geosciences, China; State Key Laboratory of Isotope Geochemistry, Guangzhou Institute of Geochemistry, Chinese Academy of Sciences, China; CAS Center for Excellence in Deep Earth Science, China

## Abstract

High-precision U-Pb zircon ages of 125.755 ± 0.061 Ma and 124.122 ± 0.048 Ma, respectively, represent the onset and termination of Yixian Formation in the Jin-Yang basin, bracketing its duration to 1.633 ± 0.078 Myr.

The Early Cretaceous Jehol Biota, renowned for its exceptionally well-preserved volcanic-influenced ecosystem, was buried in lacustrine and occasionally fluvial sediments. This includes, notably, the Huajiying, Yixian and Jiufotang Formations in northern Hebei and western Liaoning and equivalent ash-interbedding sediments in neighboring areas [1–3] (Fig. 1). It harbors many evolutionarily significant taxonomies, e.g. feathered dinosaurs, early birds, mammals and flowering plants, representing one of the most diversified terrestrial biotas of the Mesozoic [1,2,4].

The evolutionary radiation of the Jehol Biota can be broadly divided into three phases [5], with the first phase limited to a small area in northern Hebei (Huajiying Formation), the second phase expanding to western Liaoning (Yixian Formation), marking the greatest diversification, and the third phase (Jiufotang Formation) representing the widest distribution. Accordingly, it is crucial to precisely determine the timing and duration of the Yixian Formation. Despite considerable efforts in the past two decades attempting to achieve this goal, the published results (Fig. 1D) are confusing and inadequate. (i) Despite its lowermost stratigraphic locations [3], the existing ages of the Lujiatun Unit (LJT Unit) are younger than those of the immediate overlying Lower Lava Unit. The ages of the upper Yixian Formation are younger than the overlying Jiufotang Formation. It therefore casts serious doubt on the robustness of these dating results. (ii) Although most of the published ages of the Yixian Formation, with the exception of those for the LJT Unit, define an overall decrease in age following the stratigraphic column from bottom to top, the ages within individual units do not always show a consistency in stratigraphically upward deceasing trend. These problems may stem either from inaccurate stratigraphic information of some dated samples, or from inconsistency of inter-laboratory analyses, between different dating methods (i.e. ^40^Ar/^39^Ar and U-Pb dating by either laser ablation or secondary ion probe), and relatively large analytical uncertainties, which are inadequate for the purpose of establishing a chronostratigraphic framework. We therefore use a U-Pb chemical abrasion-isotope dilution-isotope ratio mass spectrometry (CA-ID-IRMS) dating technique with a typical analytical precision <0.05% to date single zircons from volcanic tuff layers collected from the top (CY18–20), middle (CY17–17) and bottom (CY17–18 and CY17–9) of the Yixian Formation in the Jin-Yang basin (Fig. 1C), in order to tightly constrain its absolute age and duration.

The new CA-ID-IRMS ages show significant improvement in analytical precision compared with literature data (Fig 1D and E). Our new ages collectively provide very tight constraints on the onset at 125.755 ± 0.061 Ma (CY17–9) and termination at 124.122 ± 0.048 Ma (CY18–20) of the Yixian Formation, respectively, bracketing its duration to 1.633 ± 0.078 Myr. It is significantly shorter than the previous broad range estimates of ∼2–7 Myr [6]. The fossil preservation in the LJT Unit is often referred to as the ‘Chinese Pompeii’ for dinosaurs and other fossils, due to rapidly deposited catastrophic pyroclastic flows [7]. Our new age constraints with the extraordinarily short duration of the LJT Unit (<71 ± 86 Kyr) support the sudden nature of the deposition event(s) that preserved fossils in three-dimensional structures with gestures.

Some argue that the Jianshangou (JSG) and LJT Units are stratigraphically equivalent primarily based on prior chronological data [8]. However, the two units show considerably different petrographic facies, mineralogical characteristics and geochemical compositions (Supplementary Texts 1 and 2), suggesting that the two units represent separate depositional events. The difference in the two units is further confirmed by the younger age of CY17–17 from the JSG Unit than that of the LJT Unit. The ages of the LJT and JSG Units are resolvable given the extraordinary precision achieved in this study, with the JSG Unit being sequentially deposited later than the LJT Unit, in agreement with their stratigraphic relationship observed in the field and cores [3].

**Figure 1.**
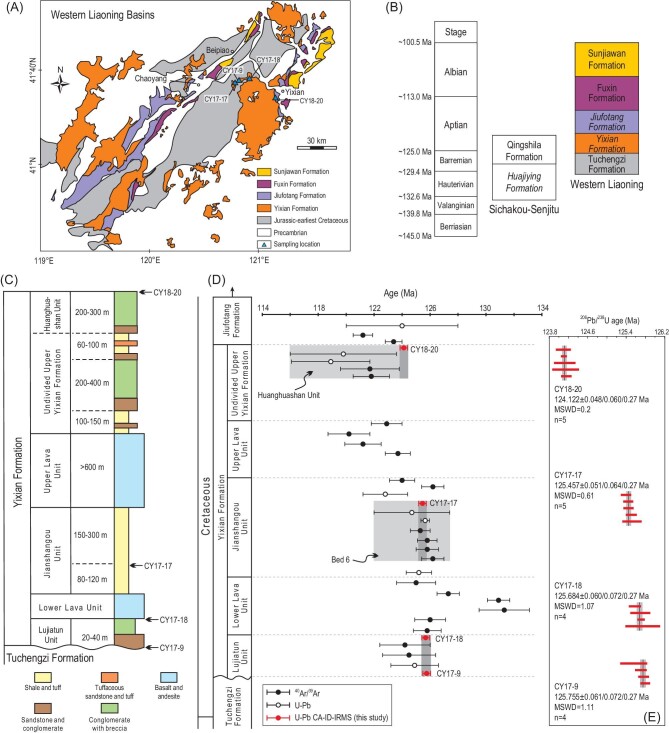
(A) The schematic geological map of western Liaoning Province (modified from Wang *et al.* [3]) with the sampling locations. (B) Early Cretaceous stratigraphic columns in northern Hebei and western Liaoning. Three formations, in italics, namely Huajiying, Yixian and Jiufotang Formations, are the main hosts of the Jehol Biota. (C) Composite stratigraphic column (modified from Zhou *et al.* [4]) and the sampling horizons of the Yixian Formation. (D) A summary plot comparing literature age data (Supplementary Table S1) with our new U-Pb CA-ID-IRMS results for the Yixian Formation. The ^40^Ar/^39^Ar dates are corrected using the decay constant of Renne *et al.* [9] and all reported uncertainties are in 2σ. Red circles denote U-Pb ages obtained in this study, with the full systematic uncertainties (uncertainty Z) for comparison with ^40^Ar/^39^Ar dates. (E) Ranked-age plots for single zircon U-Pb analyses for CY18–20, CY17–17, CY17–18 and CY17–9.

The refined duration of the Yixian Formation also yields important insights on the duration of the JSG lacustrine deposits. The sedimentary cyclicity was interpreted as periodic lake-level fluctuations plausibly caused by climatic changes that in turn may be orbitally forced Milankovitch cycles [3,10]. If so, our study effectively rules out the lacustrine cyclostratigraphy documented in part of the Yixian Formation being driven by orbital eccentricity, but more likely obliquity or precession signals. Our new data indicate that the entire Yixian Formation is only 1.633 ± 0.078 Myr maximum, which means that the JSG Unit within the Yixian Formation should be <1.633 Myr. This would clearly exclude the possibility of interpreting the 2 m cycle as a 100 Kyr eccentricity cycle for a 41 m JSG Unit. It is possible that the sedimentation rates of lacustrine environments between the studied outcrops and drill cores are highly variable, and obtaining accurate Milankovitch cycle signals from the terrestrial sediments remains a challenging goal without further high-resolution geochronological constraints.

## Supplementary Material

nwab063_Supplemental_File
