# Supplementary material for: High-precision geochronological constraints on the duration of ‘Dinosaur Pompeii’ and the Yixian Formation
Source: Natl Sci Rev. 2021 Apr 12;8(6):nwab063. doi: 10.1093/nsr/nwab063 (PMC8288181; doi:10.1093/nsr/nwab063)
Supplement: nwab063_Supplemental_File [file nwab063_supplemental_file.pdf]

High Precision Geochronological Constraints on the Duration of 'Dinosaur Pompeii' and the Yixian Formation

Supporting Figures S1-S15

Supplementary Texts 1-3

Supplementary Tables S1-S4

Supporting Figures

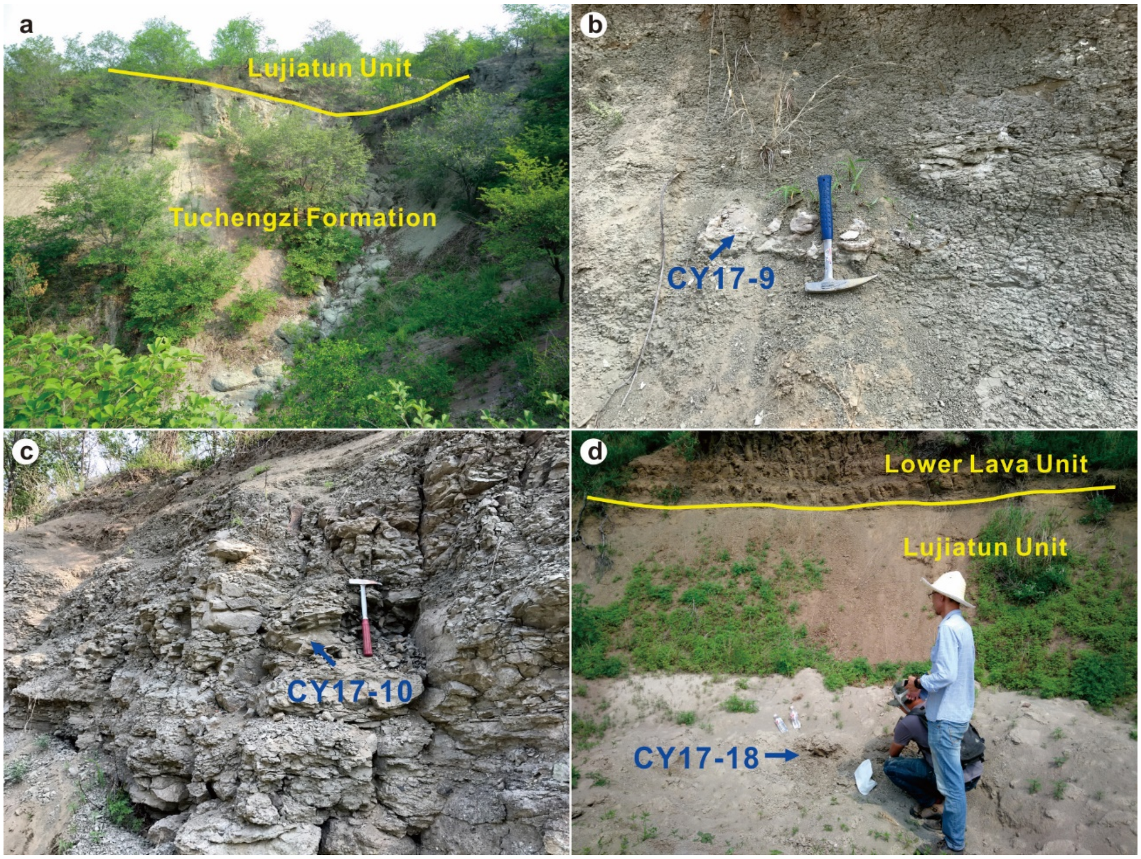

Fig. S1. Field photographs showing the sampling locations from LJT Unit. (a) The contact between Tuchengzi Formation and LJT Unit of Yixian Formation (41°36'1.6"N, 120°48'57.1"E). (b) Field appearance of sample CY17-9, at the bottom of LJT Unit. (c) Field appearance of sample CY17-10, at the lower part of LJT Unit. (d) The contact between LJT and LL units (Yixian Formation), and the field appearance of sample CY17-18.

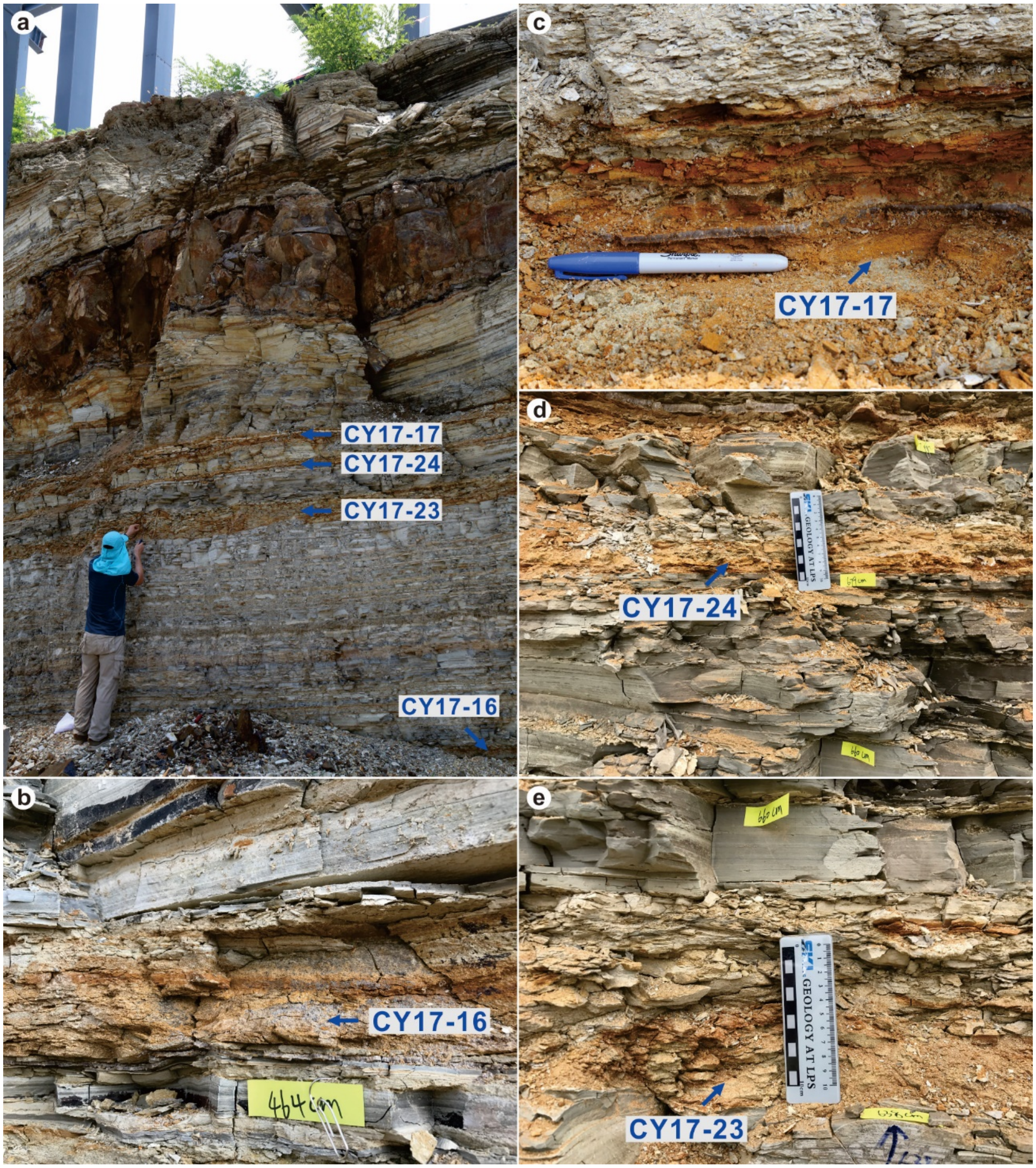

Fig. S2. Field photographs showing the horizons of sample collection at JSG Unit. (a) Overview image of the JSG Unit at the Sihetun Museum. Close-up view of the samples (b) CY17-16, (c) CY17-17, (d) CY17-24, and (e) CY17-23. The yellow sticky note for scale is 1.5 cm in height. The marker in (c) for scale is 14 cm long.

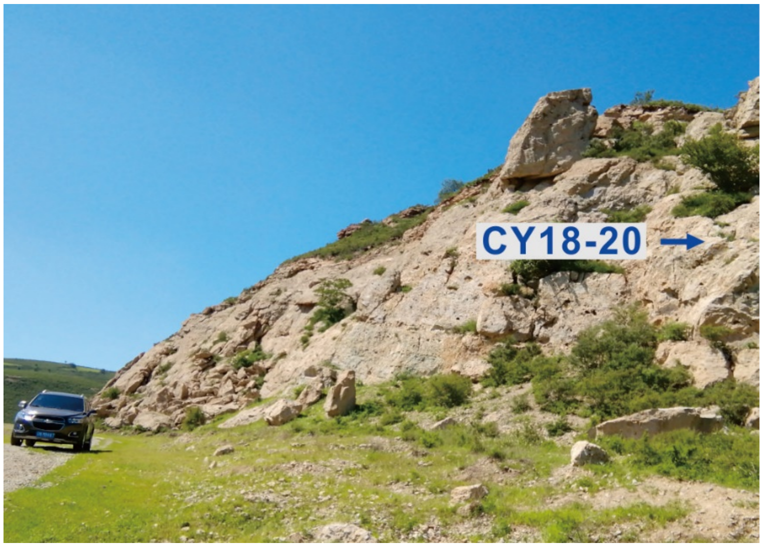

Fig. S3. Field photograph showing sample CY18-20 collected from Huanghuashan Unit.

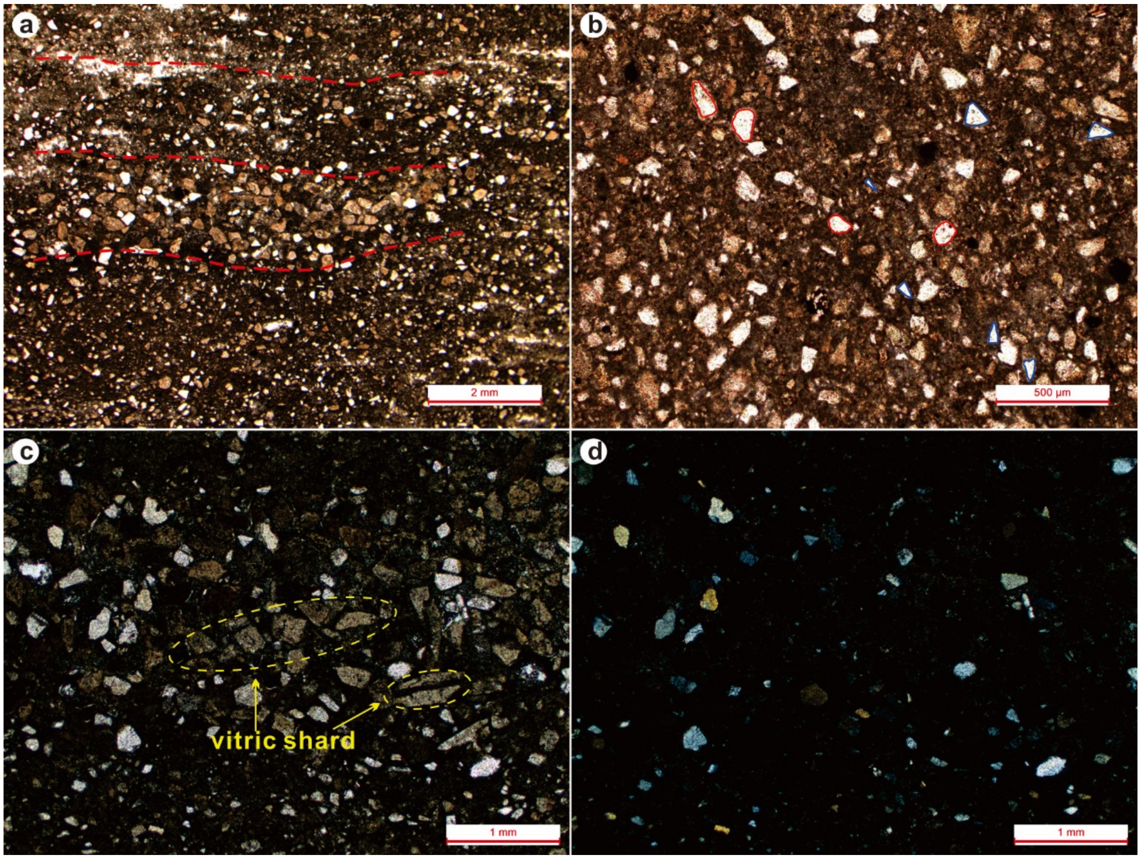

Fig. S4. Photomicrographs of sample CY17-9. (a) The overview shows the micro-bedding texture. (b) The magnified view of the lower part of (a), showing two shapes of quartz are mixed: (1) the volcanic quartz fragments (outlined in blue) are angular, most of them are smaller than 100 µm; (2) the sedimentary quartz fragments (red circle) are well rounded (~ 100 µm), which represent the addition of terrigenous detrital fractions during the deposition. (c) The close-up view shows vitric shard (0.2-0.6mm long), goes complete extinction under the cross-polarized light (d). (a), (b), and (c) are taken under plane-polarized light, (d) is taken under cross-polarized light.

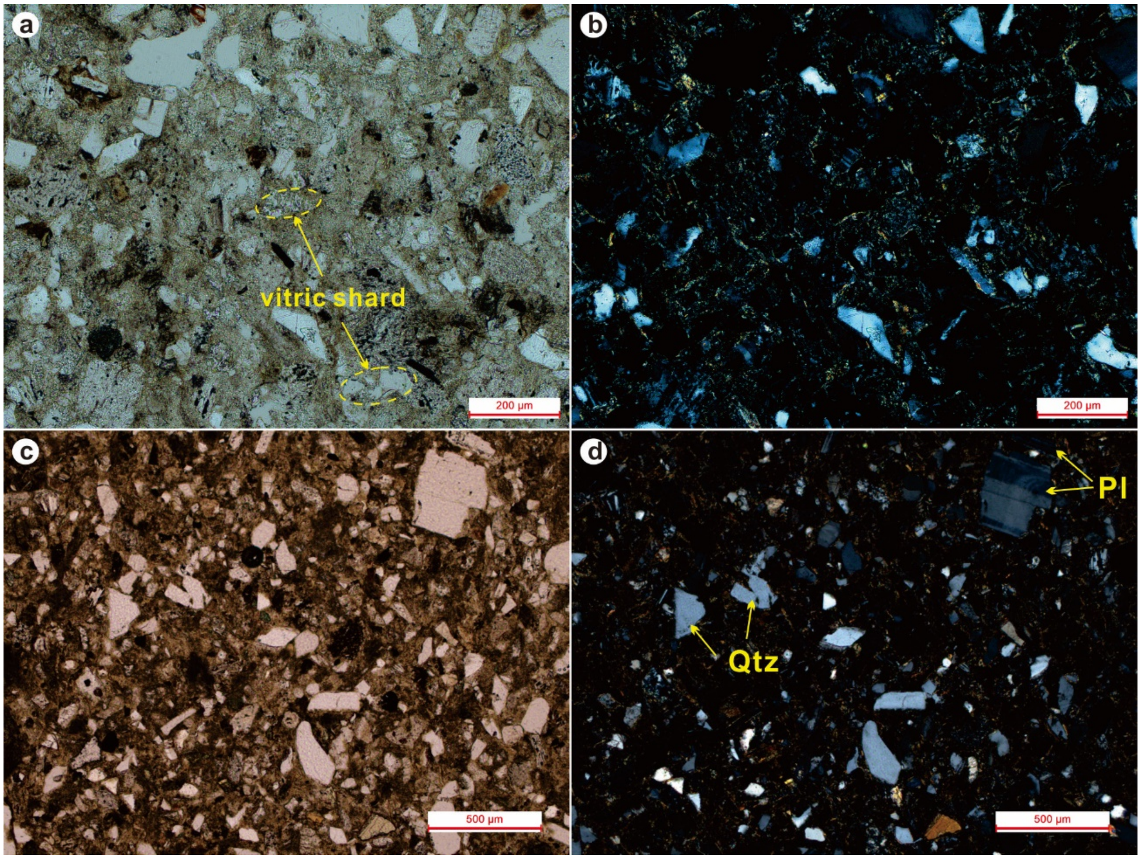

Fig. S5. Photomicrographs of sample CY17-10. (a) and (c) are taken under plane-polarized light, (b) and (d) are taken under cross-polarized light. Pl: plagioclase; Qtz: quartz.

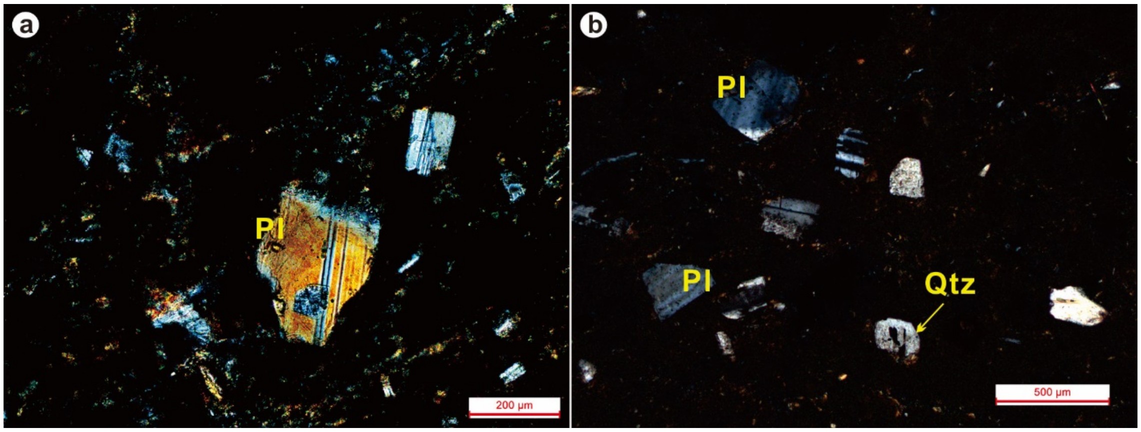

Fig. S6. Photomicrographs of sample CY17-18. All the images are taken under cross-polarized light. Pl: plagioclase; Qtz: quartz.

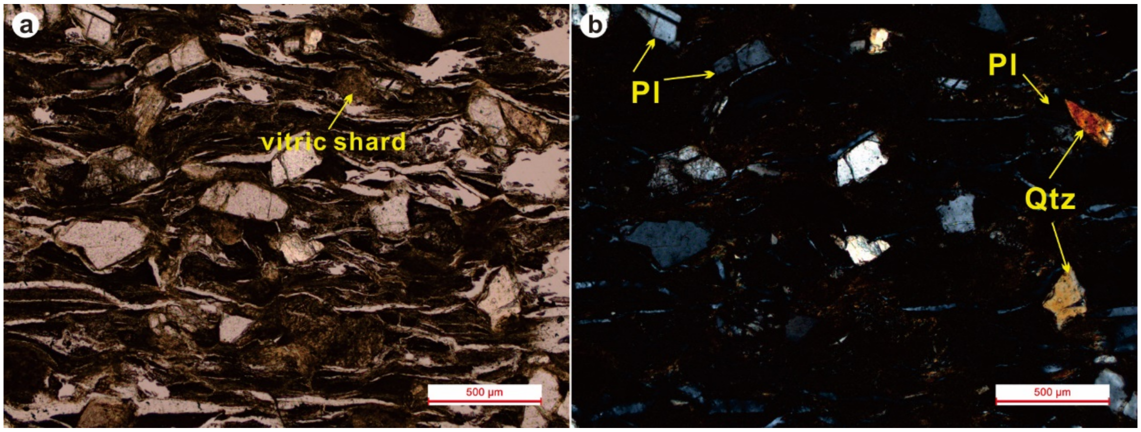

Fig. S7. Photomicrographs of sample CY17-16. (a) is taken under plane-polarized light, and (b) is taken under cross-polarized light. Pl: plagioclase; Qtz: quartz.

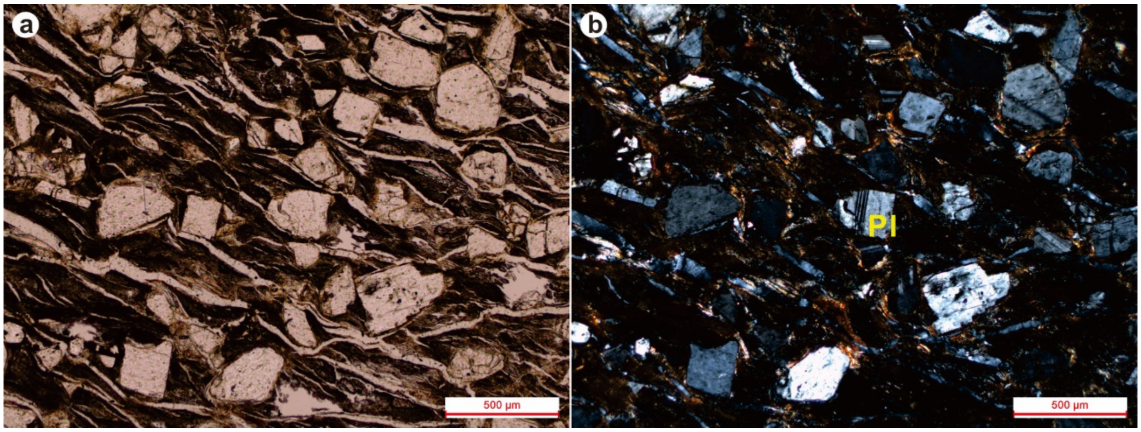

Fig. S8. Photomicrographs of sample CY17-17. (a) is taken under plane-polarized light, and (b) is taken under cross-polarized light. Pl: plagioclase.

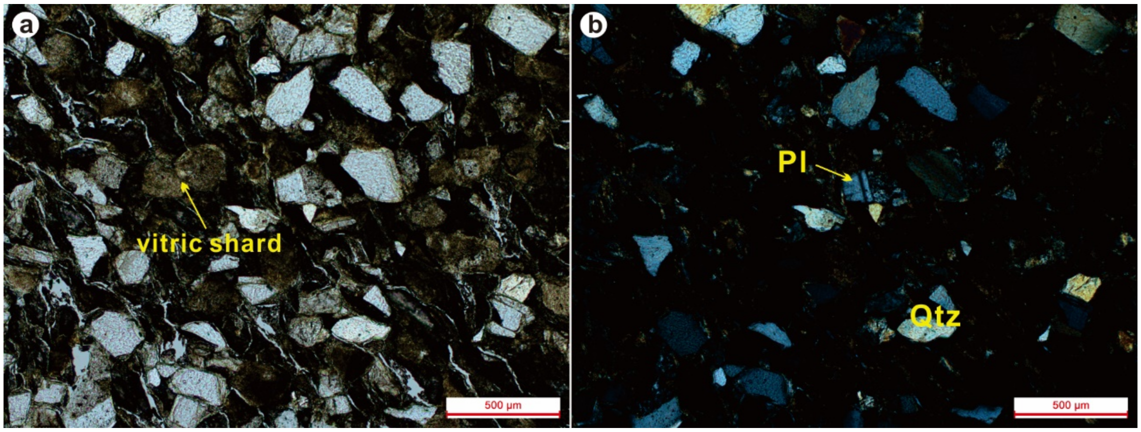

Fig. S9. Photomicrographs of sample CY17-23. (a) is taken under plane-polarized light, and (b) is taken under cross-polarized light. Pl: plagioclase; Qtz: quartz.

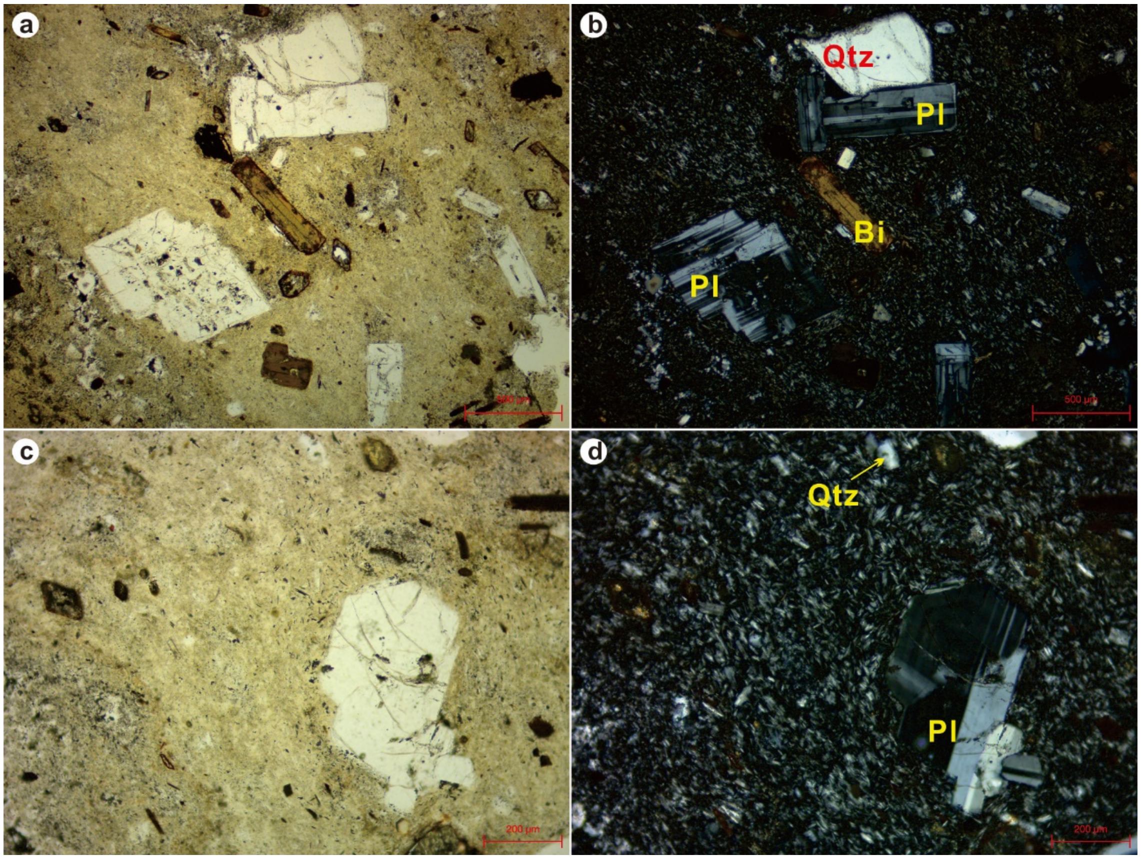

Fig. S10. Photomicrographs of sample CY18-20. (a) and (c) are taken under plane-polarized light, (b) and (d) are taken under cross-polarized light. Pl: plagioclase; Qtz: quartz; Bi: biotite.

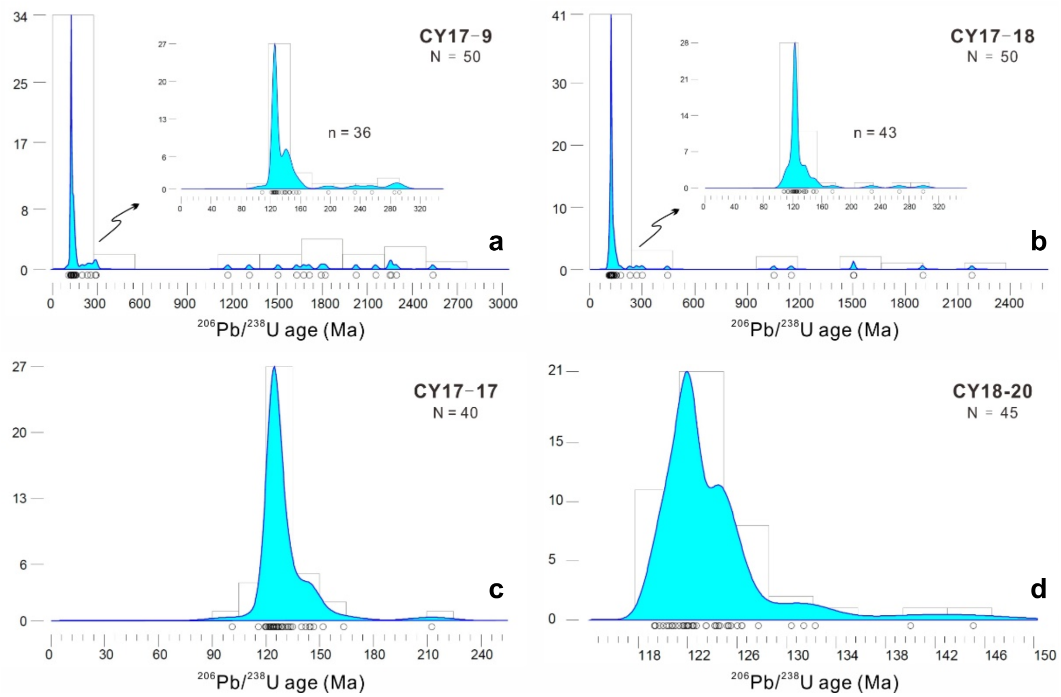

Fig. S11. Kernel density estimation *(1)* of zircons analyzed by laser ablation ICP-MS for samples (a) CY17-9, (b) CY17-18, (c) CY17-17, and (d) CY18-20. All the analyzed zircons are included, regardless of concordance. These results were used as a guide to select the youngest population for higher precision U-Pb dating by CA-ID-IRMS method.

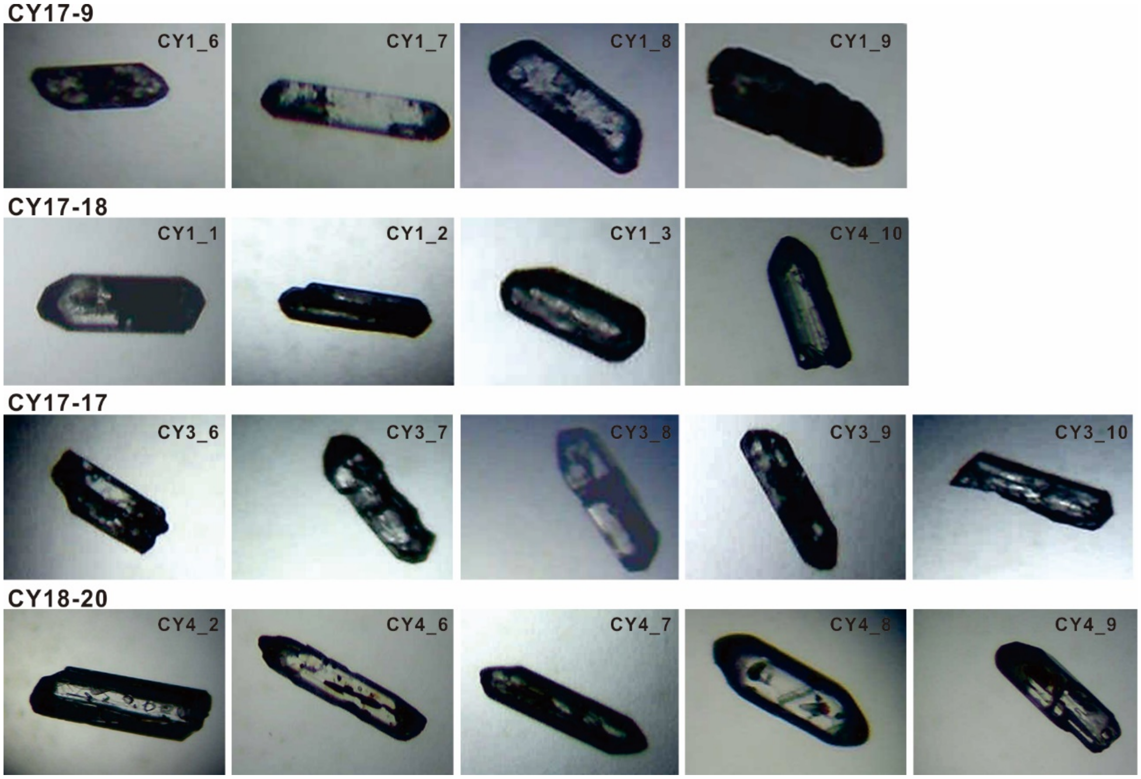

Fig. S12. Images of single zircon grains after leaching procedure (chemical abrasion) for each sample (CY17-9, CY17-18, CY17-17, and CY18-20) that were analyzed by CA-ID-IRMS.

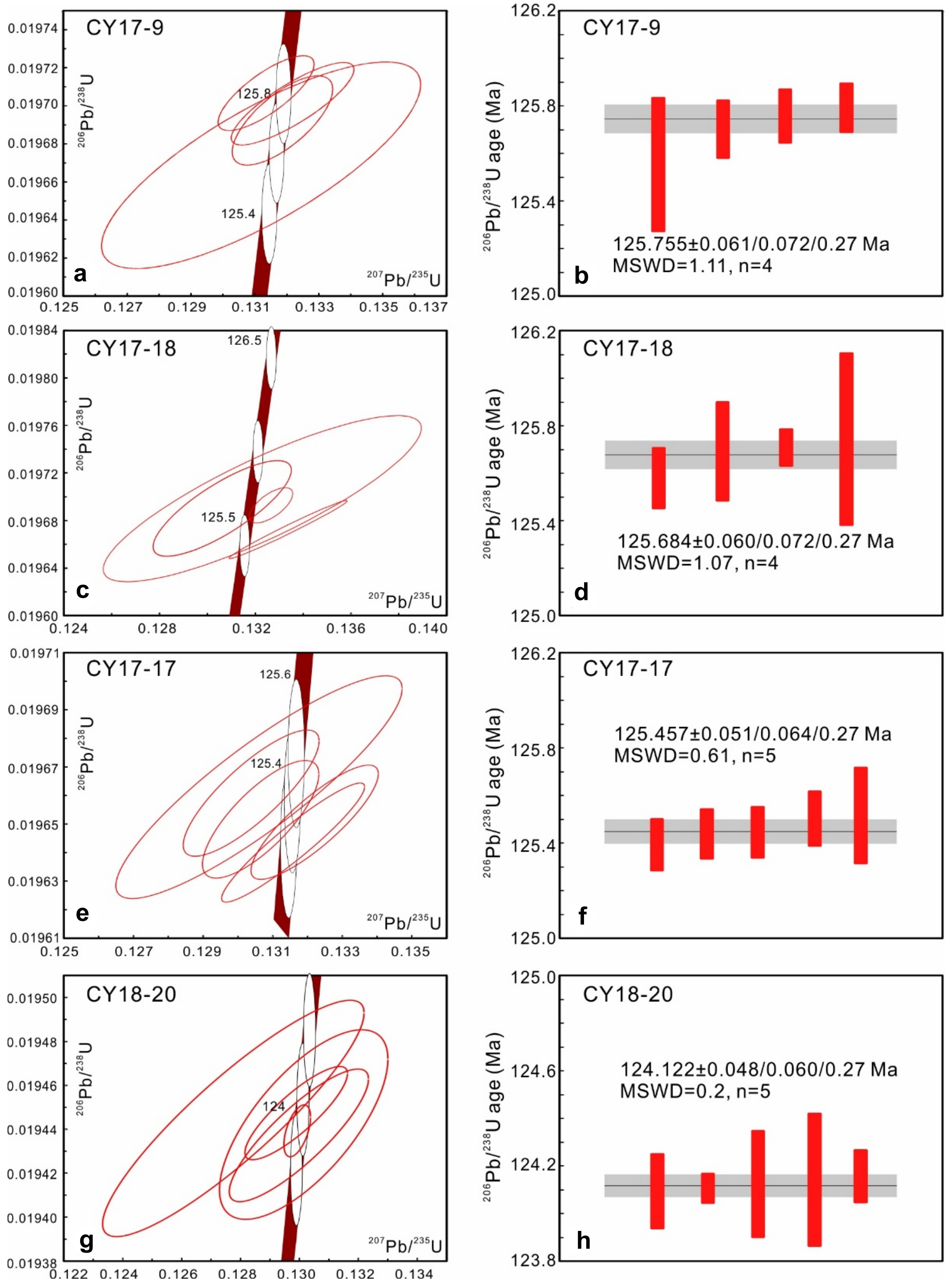

Fig. S13. Concordia diagrams (a, c, e, g) and ranked-age plots (b, d, f, h) show the results from analyzed samples by CA-ID-IRMS. The red color band in the Concordia plots represents the decay constant uncertainty. In the ranked-age plots, each bar represents the date and analytical uncertainty of an individual zircon grain and the grey bar showing the weighted average (95% uncertainty) of all analyzed zircon grains for each sample.

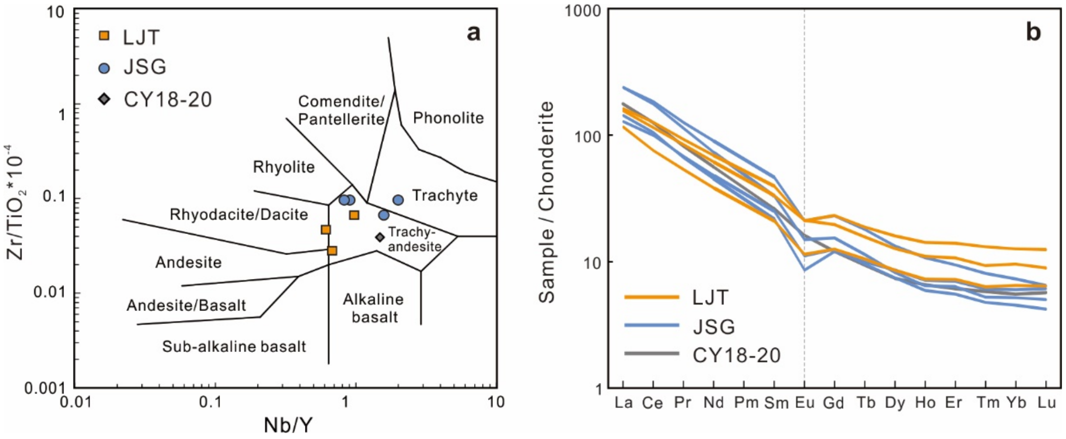

Fig. S14. (a) Nb/Y-Zr/TiO_2 classification diagram *(2)*. (b) Diagram of chondrite-normalized rare earth element (REE) patterns. Normalization values are after Sun and McDonough, *(3)*.

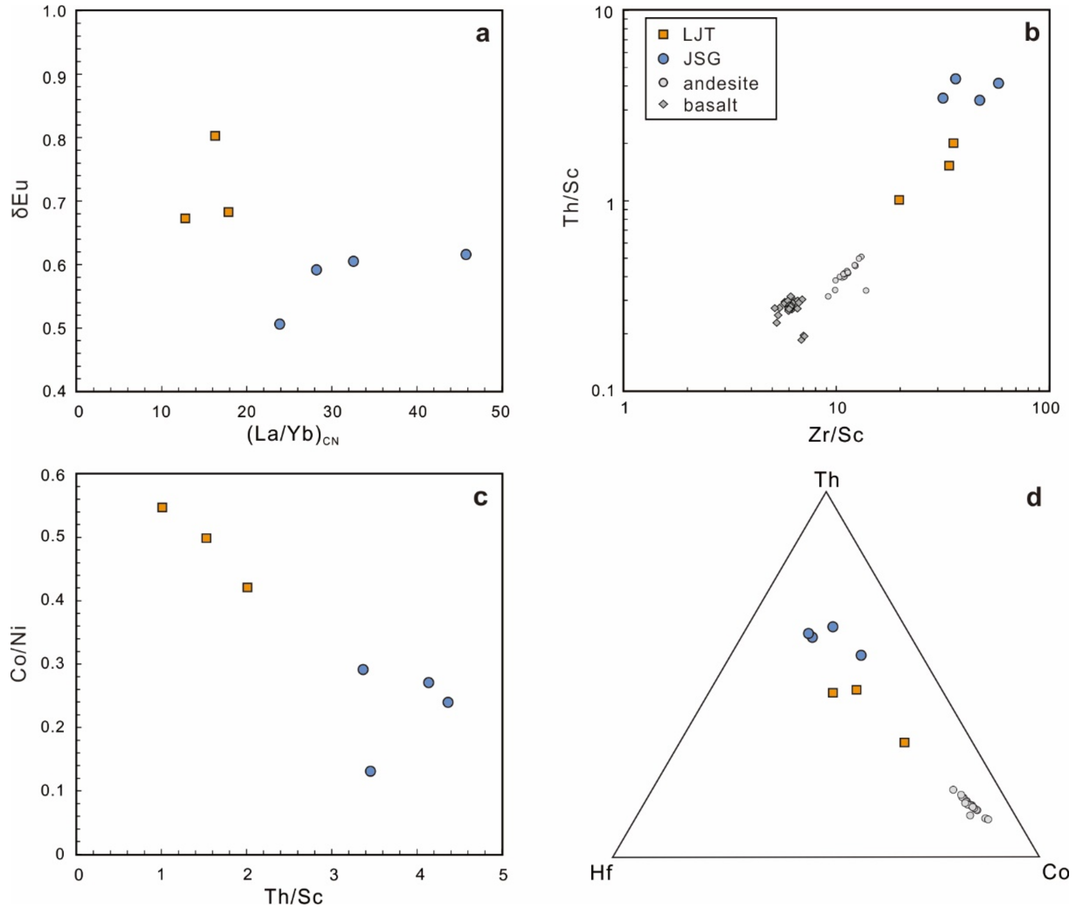

Fig. S15. Plots of (a) $(La/Yb)_{CN}$-δEu, (b) Zr/Sc-Th/Sc *(4)*, (c) Th/Sc-Co/Ni, and (d) Th-Hf-Co ternary diagrams *(5)*. Also shown in (b) and (d) are the andesites and basalts from Sihetun area, which are compiled from Wang, et al. *(6)*, Gao, et al. *(7)*, Yang and Li, *(8)*.

References

1. P. Vermeesch, On the visualisation of detrital age distributions. Chem. Geol. 312, 190–194 (2012).
2. J. A. Winchester, P. A. Floyd, Geochemical discrimination of different magma series and their differentiation products using immobile elements. Chem. Geol. 20, 325–343 (1977).
3. S. S. Sun, W. F. McDonough, Chemical and Isotopic Systematics of Oceanic Basalts: Implications for Mantle Composition and Processes. Geol. Soc. Spec. Pub. 42, 313–345 (1989).
4. S. R. Taylor, S. M. McLennan, The continental crust: its composition and evolution. Black Scientific Publications, pp. 1–312 (1985).
5. S. M. McLennan, S. Hemming, D. K. McDaniel, G. N. Hanson, Geochemical approaches to sedimentation, provenance, and tectonics, in Johnson, M.J., and Basu, A., eds., Processes Controlling the Composition of Clastic Sediments. Geol. Soc. Spec. Paper 284, 21–40 (1993).
6. X. R. Wang, S. Gao, X. M. Liu, H. L. Yuan, Z. C. Hu, H. Zhang, X. C. Wang, Geochemistry of high-Mg andesites from the early Cretaceous Yixian Formation, western Liaoning: Implications for lower crustal delamination and Sr/Y variations. Sci. China D 49, 904–914 (2006).
7. S. Gao, R. L. Rudnick, W. L. Xu, H. L. Yuan, Y. S. Liu, R. J. Walker, I. S. Puchtel, X. M. Liu, H. Huang, X. R. Wang, J. Yang, Recycling deep cratonic lithosphere and generation of intraplate magmatism in the North China Craton. Earth Planet. Sc. Lett. 270, 41-53 (2008).
8. W. Yang, S. G. Li, Geochronology and geochemistry of the Mesozoic volcanic rocks in Western Liaoning: Implications for lithospheric thinning of the North China Craton. Lithos 102, 88-117 (2008).

Supplementary Text 1

Samples

Yixian Formation

Underlain by the Tuchengzi Formation and overlain by the Jiufotang Formation, the Yixian Formation is mainly composed of andesites, andesite-breccia, agglomerates, basalts, and the fossil-bearing sedimentary intercalations rich in tuffaceous materials *(1)*. It comprises five units, in an ascending order, Lujiatun (LJT for short), Lower Lava, Jianshangou (JSG for short), Upper Lava units, and undivided Upper Yixian Formation. Among them, LJT and JSG units are the principle hosts of the fossils of the Jehol Biota. The LJT Unit, situated at the base of the Yixian Formation *(2)*, consists of massive tuffaceous sandstone or siltstone *(3)*. Numerous three-dimensionally preserved vertebrate fossils have been recovered, including some specimens preserving behavioral information but with no traces of soft tissues *(4,5)*. For example, the *Mei long* holotype is preserved in a remarkable life tuck-in sleeping (or resting) posture *(5)*. Pyroclastic flows are hypothesized as a potential cause for the mass mortality events and the remarkable preservation *(3)*. In contrast, the JSG Unit is mainly composed of fine laminated sediments and yields abundant flattened vertebrates usually with various soft tissues preserved, e.g., skins, hairs, feathers *(1,6,7)*. For instance, the *Sinosauropteryx prima* has interesting integumentary structures that could provide information about the origin of feathers *(1)*.

Field observations and sampling horizons

Lujiatun Unit. The Lujiatun (LJT for short) Unit is the lowermost unit of the Yixian Formation overlain by the Lower Lava (LL for short) Unit (Figs. S1a and 1d), which is mainly composed of volcanic conglomerates, massive tuffaceous sandstone or siltstone. LJT Unit generally shows little to no stratification *(8,9)*. Three samples have been collected from the LJT Unit. CY17-9 was collected from the bottom of LJT Unit (41°35'59.8"N, 120°48'59.8"E) (Fig. S1b). CY17-10 was collected along the same section as CY17-9, representing the lower part of LJT Unit (41°35'57"N, 120°49'3.1"E). Both of them are grayish white in color (Figs. S1b-c), dense and hard. CY17-18 was collected from the topmost of the LJT Unit (41°36'7.6"N, 120°54'47.5"E), which is about 1.8 meters

below the contact between LJT and LL units (Fig. S1d). CY17-18 is off-white in-color and poorly consolidated.

Jianshangou Unit. Samples were collected from Jianshangou (JSG for short) Unit at the Sihetun section (41°35'18.93"N, 120°47'32.66"E). As show in Fig. S2a, at least 15 layers (h > 1 cm) of yellowish tuff were identified within the 4.6-meters thickness section, and all of the tuffs are poorly consolidated. Four tuff samples were collected, CY17-16, CY17-23, CY17-24, and CY17-17 in ascending order and the sampled horizons shown in Fig. S2a. The thicknesses for the sampled tuff layers are 7cm, 14cm, 7cm, and 7cm, respectively. Fossils in the JSG Unit were mainly found below CY17-17 which was dated in this study.

Huanghuashan Unit. The stratigraphy of the Upper Yixian Formation remains controversial. Several units, namely Huanghuashan Unit, Jingangshan Unit, Dawangzhangzi Unit, have been proposed, but the temporal relationship between these units is not assessed yet. Instead of going into specific debates, the Upper Yixian Formation is simply treated as undivided in this study. Nevertheless, the Huanghuashan Unit is considered as on the top of the uppermost fossiliferous unit of the Yixian Formation *(10-12)*, because it is in conformable contact with the overlying Jiufotang Formation. It thus represents the dating target for the termination of the Yixian Formation. Sample CY18-20 was collected at Songbahu section (Fig. S3; 41°28'10.97"N, 121°10'9.72"E).

Petrography of the samples

Lujiatun Unit. The CY17-9 is a poorly to moderately sorted vitric-crystal tuff with angular to sub-rounded grains of quartz and plagioclase feldspar (Fig. S4). There are abundant sickle-shaped vitric fragments, and most of them have been devitrified (Figs. S4c-d). Considering the mixtures of sedimentary quartz and volcanic quartz (Fig. S4b), it is reasonable to classify it as a sedimentary tuff.

Similar to CY17-9, the CY17-10 is a poorly sorted vitric-crystal tuff (Fig. S5). The crystal fragments are dominated by angular to subangular quartz (>20 – 100 μm), plagioclase feldspar (>50 – 200 μm), and small amounts of biotite. The quartz fragments are often with magmatic corrosion gulfs. Some chicken bone-shaped vitric fragments are observed.

The CY17-18 is a poorly consolidated tuff with small amount of crystal fragments (Fig. S6). Plagioclase (~100 – 350 μm) is white-colored with perfect cleavage and albite twin. The quartz fragments (<100 μm) are often with magmatic corrosion gulfs. Clasts float in a clayey groundmass of devitrified volcanic glass and very fine ashes.

Jianshangou Unit. All samples collected from JSG Unit are poorly consolidated tuff. During the preparation of thin-section, the textural integrity of the samples were compromised. The descriptions here are therefore only referring to the components.

The CY17-16 is a crystal tuff with plate-like plagioclase (>100 – 250 μm) and small amounts of angular quartz (up to 200 μm) (Fig. S7). Some orange colored vitric shard are observed.

The CY17-17 is a crystal tuff with abundant angular to subangular plagioclase and quartz (both are 100 – 200 μm) (Fig. S8).

The CY17-23 is a poorly consolidated crystal tuff. The crystal fragments include angular to subangular quartz and plagioclase feldspar (both are 100 – 200 μm) (Fig. S9). Some vitric shard and altered plagioclase are observed. In summary, samples CY17-23 and CY17-17 are crystal tuffs and sharing similar compositions.

Huanghuashan Unit. The sample CY18-20 is greyish in color and has a porphyritic texture (Fig. S10). The predominant phenocrysts are plagioclase (~10 vol%), biotite (~5 vol%), and quartz (~3 vol%). Plagioclase and biotite (both ~500 μm) occur as euhedral grains. The groundmass consists of fine-grained quartz and lath-shaped plagioclase (Fig. S10). Minor opaque minerals and zircon grains occur in the groundmass. Geochemical analyses suggest it is a trachyandesite (see Section 'Major and trace elemental compositions' below).

References

1. P. J. Chen, Z. M. Dong, S. N. Zhen, An exceptionally well-preserved theropod dinosaur from the Yixian Formation of China. Nature 391, 147–152 (1998).
2. Y. Q. Wang, P. E. Olsen, J. G. Sha, X. G. Yao, H. Y. Liao, Y. H. Pan, S. Kinney, X. L. Zhang, X. Rao, Stratigraphy, correlation, depositional environments, and cyclicity of the Early Cretaceous Yixian and ?Jurassic-Cretaceous Tuchengzi formations in the Sihetun area (NE China) based on three continuous cores. *Palaeogeogr Palaeoclimatol Palaeoecol* 464, 110–133 (2016).
3. B.Y. Jiang, G. E. Harlow, K. Wohletz, Z. H. Zhou, J. Meng, New evidence suggests pyroclastic flows are responsible for the remarkable preservation of the Jehol biota. *Nat Commun* 5, 1–7 (2014).
4. X. Xu, Y. N. Cheng, X. L. Wang, An unusual oviraptorosaurian dinosaur from China. *Nature* 417, 291–293 (2002).
5. X. Xu, M. A. Norell, A new troodontid dinosaur from China with avian-like sleeping posture. *Nature* 431, 838–841 (2004).
6. Z. H. Zhou, The origin and early evolution of birds: discoveries, disputes, and perspectives from fossil evidence. *Naturwissenschaften* 91, 455–471 (2004).
7. F. C. Zhang, S. L. Kearns, P. J. Orr, M. J.Benton, Z. H. Zhou, D. Johnson, X. Xu, X. L. Wang, Fossilized melanosomes and the colour of Cretaceous dinosaurs and birds. *Nature* 463, 1075–1078 (2010).
8. Z. H. Zhou, P. M. Barrett, J. Hilton, An exceptionally preserved Lower Cretaceous ecosystem. Nature 421, 807–814 (2003).
9. B. Y. Jiang, J. G. Sha, Preliminary analysis of the depositional environments of the Lower Cretaceous Yixian Formation in the Sihetun area, western Liaoning, China. *Cretac. Res*. 28, 183–193 (2007).
10. W. L. Wang, L. J. Zhang, S. L. Zheng, Y. Zheng, H. Zhang, Z. T. Li, F. L. Yang, A new study on the stratotype and biostratigraphy of the Yixian Stage in the Yixian-Beipiao region, Liaoning-Establishment and study of the stratotype of the Yixian Stage (in Chinese with English abstract). *Acta Geol. Sin.* 78, 433–447 (2004).
11. H. Zhang, F. L. Yang, L. J. Zhang, Z. T. Li, W. L. Wang, B. Ma, W. G. Sun, S. L. Zheng, Y. J. Zheng, New progress in the study of Huanghuashan Breccia in Yixian county, western Liaoning (in Chinese with English abstract). *Acta Geol. Sin.* 25, 321–326 (2004).
12. H. Zhang, X. M. Liu, W. Chen, Z. T. Li, F. L. Yang, The age of the top of the Yixian Formation in the Beipiao-Yixian area, western Liaoning, and its importance (in Chinese with English abstract). *Geol. China* 32, 596–603 (2005).

Supplementary Text 2

Methods and Results

Zircon U-Pb geochronology

High-precision zircon U-Pb analyses using chemical abrasion-isotope dilution-isotope ratio mass spectrometry (CA-ID-IRMS) were performed on selected crystals (Fig. S12) at the University of California, Davis. The term IRMS is used here to reflect the combined approach of both thermal ionization mass spectrometry (TIMS) for Pb isotope analyses and multi-collection (MC)-ICP-MS for U isotopic measurements. Detailed methods for CA-ID-IRMS analysis following Mattinson *(1)* can be found in Zhou, et al. *(2, 3)*, and Lan, et al. *(4)*. Zircon grains were annealed first at 900°C and subsequently analyzed by laser ablation ICP-MS for U-Pb geochronology. These were performed to identify any detrital or inherited components and to select the youngest population of zircons for the subsequent, time-consuming and labor intensive CA-ID-IRMS analysis.

Subsequently, selected grains were leached for 15 h at 190°C, and a ^{202}Pb-^{205}Pb-^{233}U-^{236}U tracer *(5)* was added to the abraded zircons prior to dissolution. Pb and U were separated from the matrix elements by standard HCl ion exchange chemistry *(6)* and Pb was loaded onto zone refined Re filaments with a silica gel activator *(7)*. Pb was analyzed on a *Triton Plus* TIMS (thermal ionization mass spectrometer) at UC Davis. Two measurement protocols were used depending on sample size. CY1_3 and CY4_10 were measured on the secondary electron multiplier (SEM) in peak jumping mode. All other samples were measured with ^{204}Pb in the axial secondary electron multiplier while all other isotopes were collected in Faraday cups coupled with 10^{13} Ω resistor amplifier boards. The gain calibration for sample ID starting with CY1 and CY3 followed a protocol described by Trinquier *(8)*, see also Zhou et al. *(2)*. For batch CY4, gain calibration was done with a relay matrix board. The SEM yield was calibrated daily and in batch CY4 confirmed with additional measurement of ^{205}Pb in the axial SEM. U isotope dilution measurements were performed on a *Neptune Plus MC-ICP-MS* (Multi-collector inductively-coupled plasma mass spectrometer) at UC Davis using an ESI APEX introduction system. Faraday cups measuring ^{238}U and ^{235}U were coupled with 10^{12} Ω resistors and ^{233}U and ^{236}U were coupled with 10^{11} Ω resistors. Measurements were corrected for instrumental mass dependent fractionation using an exponential law based on the known ^{233}U-^{236}U ratio of IRMM-3636 *(9)*. The ^{238}U/^{236}U ratio of the samples was additionally corrected using the known ^{238}U/^{235}U ratio of CRM 112a of 137.837 *(10)* using a linear correction based on sample-standard bracketing. Total procedural blank for U is 0.1 pg. All non-radiogenic

Pb was attributed to laboratory contamination. U-Pb ages and uncertainties were calculated using the algorithms of Schmitz and Schoene *(11)*. We report three levels of uncertainty for the age data, representing analytical uncertainty only (X), analytical and tracer calibration uncertainties (Y), and analytical and tracer and decay constant uncertainties (Z), respectively *(12)*. For U-Pb age comparison within the same laboratory, the first uncertainty is sufficient. To compare U-Pb dates across different laboratories, the second uncertainty should be used. When comparing dates across different methods (e.g., U-Pb *vs*. Ar-Ar dates), the third uncertainty should be used. Weighted averages and concordia diagrams were produced using Isoplot *(13)* (Figs. 4 and S13).

The zircons from samples (CY17-9 and CY17-18) show similar features. Rounded and prismatic grains are both present in those samples. The laser ablation ICP-MS analyses confirmed the presence of detrital zircon component. Some analyses are slightly discordant due to common Pb on the surface of unpolished grains, and thus do not provide reliable age information. Concordant dates ranged in age from ~2.5 Ga to 121 Ma, and ~2.2 Ga to ~120 Ma, respectively (Figs. S11a-b). For the sample CY17-9 which was collected from the bottom of the LJT Unit, CA-ID-IRMS of four selected grains (Fig. S12) overlap within error and yield a weighted-mean $^{206}Pb/^{238}U$ age of 125.755 ± 0.061/0.072/0.27 Ma (n = 4, mean square weighted deviation [MSWD] = 1.11) (Fig. S13a-b). Similarly, for the sample CY17-18 which was collected from the top of the LJT Unit, four zircon grains yield a weighted-mean $^{206}Pb/^{238}U$ age of 125.684 ± 0.060/0.072/0.27 Ma (n = 4, MSWD = 1.07) (Fig. S13c-d).

The zircon grains from two tuff samples (CY17-17 and CY18-20) are light yellow and transparent in color, most of the grains are prismatic (Fig. S12). LA-ICP-MS analyses revealed a minor detrital component of zircons ranging in age from ~212 Ma to ~120 Ma, and ~145 Ma to ~120 Ma, respectively (Figs. S11c-d). Selected single zircon grains from the youngest (~120 Ma) group were analyzed by CA-ID-IRMS. The data from sample CY17-17 are in good agreement with each other and yield a weighted-mean $^{206}Pb/^{238}U$ age of 125.457 ± 0.051/0.064/0.27 Ma (n = 5, MSWD = 0.61) (Fig. S13e-f). Five grains from CY18-20 yield a weighted-mean $^{206}Pb/^{238}U$ age of 124.122 ± 0.048/0.062/0.27 Ma (n = 5, MSWD = 0.2) (Fig. S13g-h).

The synthetic standard solution from the EARTHTIME project of 100 Ma, yielded a $^{206}Pb/^{238}U$ date of 100.120 ± 0.064 Ma (n = 4). Temora zircons yielded a $^{206}Pb/^{238}U$ date of 417.01 ± 0.24 Ma (n = 4). For the standards, Pb and U were separated in the same way as the samples and measured with the same procedures.

<u>Bulk-rock mineralogical and geochemical analyses</u>

Bulk-rock analyses have been conducted on eight samples collected from LJT, JSG and Huanghuashan units (LJT Unit: CY17-9, CY17-10, and CY17-18; JSG Unit: CY17-16, CY17-17, CY17-23 and CY17-24: Huanghuashan Unit: CY18-20). Sample chips obtained from the central parts of the rocks were handpicked and chosen for further cleaning. The chips were leached with 2% HCl and washed ultrasonically in deionized water before dried and powdered to 200 mesh in a corundum mill.

Mineral assemblages. Determinations of clay mineral assemblages were carried out on un-oriented powder mounts by X-ray diffraction (XRD; Bruker D8 Advance) at the Guangzhou Institute of Geochemistry, Chinese Academy of Sciences. Qualitative and semi-quantitative characterization of mineralogy is based on peak intensity measurements on X-ray patterns, and the diagnostic peak and correction intensity factor were obtained for each mineral. The results are listed in Table S3. The JSG samples (CY17-16, CY17-17, CY17-23 and CY17-24) are mainly composed of clay mineral (smectite; 87.7% – 94.9%), with a small amount of clastic materials. The samples contain minor quartz (0 – 1.3%) and feldspar (3.1% – 11.2%). Besides, CY17-24 contains some calcite (6.1%). In contrast, the LJT samples (CY17-9, CY17-10, and CY17-18) are mainly composed of clay minerals (illite, smectite, and its intermediate product; 25.2% – 62%), with plenty of clastic materials. Each sample contains abundant quartz and feldspar (38% – 63.4%). Additionally, CY17-9 is rich in calcite (34.4%).

Major and trace elemental compositions. Samples were analyzed for major and trace elemental compositions at the Wuhan Sample Solution Analytical Technology Co., Ltd., Wuhan, China. Major element analyses were conducted on X-ray fluorescence spectroscopy (XRF) on fused glass disks using a Rigaku Primus Ⅱ XRF instrument. A pre-ignition was used to determine the loss on ignition (LOI) prior to major element analyses. Trace element analyses were determined with Agilent 7700e ICP-MS. Samples from JSG and LJT units are characterized by a high loss on ignition (LOI) contents (2.94% – 17.49%; Table S4), consistent with the high percentage of clay minerals. The chemical compositions are characterized by high Al_2O_3 (10.00% – 17.05%), low TiO_2 (0.13% – 0.64%), MnO (0.02% – 0.41%), P_2O_5 (0.05% – 0.17%), and alkalis contents (Na_2O + K_2O; 2.90% – 6.55%). More specifically, the samples (CY17-9 and CY17-24) with the lowest SiO_2 (42.32% and 48.42%) contain the highest CaO (21.45% and 12.21%) and LOI contents (17.49% and 12.92%), which is consistent with high percentage of the calcite. By comparison, the other five samples possess higher SiO_2 (57.92% – 66.98%) and lower CaO contents (2.75% – 3.03%). The Nb/Y-Zr/TiO_2 classification diagram is utilized, since all the selected elements are generally considered to remain immobile during alteration and weathering. All the samples fall within the range of trachyandesite to trachyte (Fig. S14a), suggesting similar original compositions.

The samples have very similar chondrite-normalized (CN; normalization values after Sun and McDonough, *(14)*) rare earth element (REE) patterns with enrichment of light REE (LREE) over heavy REE (HREE) and pronounced negative Eu anomalies (Fig. S13b). Specifically, samples from JSG Unit display a more fractionated REE pattern [$(La/Yb)_{CN}$ = 23.91 – 45.76] and stronger negative Eu anomalies [δEu = 0.51 – 0.62; $\delta Eu = Eu_{CN}/(Sm_{CN} + Gd_{CN})^{1/2}$] than that of the samples from LJT Unit [$(La/Yb)_{CN}$ = 12.80 – 17.88; δEu = 0.67 – 0.80] (Figs. S14b and 15a).

Some elements such as Sc, Th, U, Co and Cr are known to be an indicator for source composition *(15)*. For instance the Th/Sc ratio is considered unaffected by sedimentary processes *(16)* and represents a useful indicator of source rocks. Thus, the diagnostic parameters, i.e., Th/Sc, Zr/Sc, and Co/Ni are selected as proxies for the source characterization. The JSG and LJT samples, as well as the andesites and basalts from the same units *(17-19)*, form a linear correlation in the Zr/Sc-Th/Sc diagram (Fig. S15b). The plot of Th/Sc *versus* Co/Ni distinguishes between JSG and LJT samples, as the former are characterized by higher Th/Sc and lower Co/Ni ratios (Fig. S15c). In addition, the JSG and LJT samples fall into different areas in the Th-Hf-Co ternary diagram, which is commonly used to discriminate the provenance of sedimentary rocks *(15)* (Fig. S15d).

References

1. J. M. Mattinson, Zircon U–Pb chemical abrasion ("CA-TIMS") method: Combined annealing and multi-step partial dissolution analysis for improved precision and accuracy of zircon ages. *Chem. Geol.* 220, 47–66 (2005).
2. C. M. Zhou, M. H. Huyskens, X. G. Lang, S. H. Xiao, Q. Z. Yin, Calibrating the terminations of Cryogenian global glaciations. *Geology* 251, 251–254 (2019).
3. C. M. Zhou, M. H. Huyskens, S. H. Xiao, Q. Z. Yin, Refining the termination age of the Cryogenian Sturtian glaciation in South China. *Palaeoworld* 29, 462–468 (2020).
4. Z. W. Lan, M. H. Huyskens, K. Lu, X. H. Li, G. Y. Zhang, D. B. Lu, Q. Z. Yin, Toward refining the onset age of Sturtian glaciation in South China. *Precambrian Res.* 338, 105555 (2020).
5. M. H. Huyskens, S. Zink, Y. Amelin, Evaluation of temperature-time conditions for the chemical abrasion treatment of single zircons for U-Pb geochronology. *Chem. Geol.* 438, 25–35 (2016).
6. T. Krogh, A low-contamination method for hydrothermal decomposition of zircon and extraction of U and Pb for isotopic age determinations. *Geochim. Cosmochim. Ac.* 37, 485–494 (1973).
7. M. H. Huyskens, T. Iizuka, Y. Amelin, Evaluation of colloidal silicagels for lead isotopic measurements using thermal ionisation mass spectrometry. *J. Anal. Atom. Spectrom.* 27, 1439–1446 (2012).
8. A. Trinquier, Fractionation of Oxygen Isotopes by Thermal Ionization Mass Spectrometry Inferred from Simultaneous Measurement of $^{17}O/^{16}O$ and $^{18}O/^{16}O$ Ratios and Implications for the ^{182}Hf-^{182}W Systematics: *Anal. Chem.* 88, 5600–5604 (2016).
9. A. Verbruggen, A. Alonso, R. Eykens, F. Kehoe, H. Kühn, S. Richter, Y. Aregbe, Preparation and Certification of IRMM 3636, IRMM 3636a and IRMM 3636b. OPOCE, 24pp (2008).
10. S. Richter, R. Eykens, H. Kühn, Y. Aregbe, A. Verbruggen, S. Weyer, New average values for the n(^{238}U)/n(^{235}U) isotope ratios of natural uranium standards: *Int. J. Mass Spectrom.* 295, 94–97 (2010).
11. M. D. Schmitz, B. Schoene, Derivation of isotope ratios, errors, and error correlations for U-Pb geochronology using ^{205}Pb-^{235}U-^{233}U) spiked isotope dilution thermal ionization mass spectrometric data. *Geochem. Geophy. Geosy.* 8, Q08006 (2007).
12. I. M. Villa, M. L. Bonardi, P. De Bièvre, N. E. Holden, P. R. Renne, IUPAC-IUGS status report on the half-lives of ^{238}U, ^{235}U and ^{234}U: *Geochim. Cosmochim. Ac* 172, 387–392 (2016).
13. K. Ludwig, Isoplot 3.75. A Geochronological toolkit for Microsoft Excel. Berkeley Geochronology Center, *Spec. Pub.* 5 (2012).
14. S. S. Sun, W. F. McDonough, Chemical and Isotopic Systematics of Oceanic Basalts: Implications for Mantle Composition and Processes. *Geol. Soc. Spec. Pub*. 42, 313–345 (1989).
15. S. R. Taylor, S. M. McLennan, The continental crust: its composition and evolution. Black Scientific Publications, pp. 1–312 (1985).
16. S. M. McLennan, S. Hemming, D. K. McDaniel, G. N. Hanson, Geochemical approaches to sedimentation, provenance, and tectonics, in Johnson, M.J., and Basu, A., eds., Processes Controlling the Composition of Clastic Sediments. Geol. Soc. Spec. Paper 284, 21–40 (1993).
17. X. R. Wang, S. Gao, X. M. Liu, H. L. Yuan, Z. C. Hu, H. Zhang, X. C. Wang, Geochemistry of high-Mg andesites from the early Cretaceous Yixian Formation, western Liaoning: Implications for lower crustal delamination and Sr/Y variations. *Sci. China D* 49, 904–914 (2006).
18. S. Gao, R. L. Rudnick, W. L. Xu, H. L. Yuan, Y. S. Liu, R. J. Walker, I. S. Puchtel, X. M. Liu, H. Huang, X. R. Wang, J. Yang, Recycling deep cratonic lithosphere and generation of intraplate magmatism in the North China Craton. *Earth Planet. Sc. Lett.* 270, 41-53 (2008).

19. W. Yang, S. G. Li, Geochronology and geochemistry of the Mesozoic volcanic rocks in Western Liaoning: Implications for lithospheric thinning of the North China Craton. *Lithos* 102, 88-117 (2008).

Supplementary Text 3

Cyclicity of the Jianshangou lacustrine deposits

The refined duration of the Yixian Formation also yields important insights on the duration of the JSG lacustrine deposits. The sedimentary cyclicity was interpreted as periodic lake-level fluctuations plausibly caused by climatic changes that in turn may be orbitally forced Milankovitch cycles *(1,2)*. Both studies *(1,2)* observed ~2 m cycle, and ~1 m cycle, and Wu et al. *(2)* observed additional 50-67 cm and 18-42 cm higher frequency cycles based on anhysteretic remanent magnetization and magnetic susceptibility at the Sihetun section. Wang et al. *(1)* hypothesized that their ~2 m cycles reflect obliquity forcing (~37 kyr), and their ~1 m cycles reflect the climatic precession cycle (~20 kyr), whereas Wu et al. *(2)* argued that the ~2 m cycles reflect the ~100 kyr cycles (1.7 cm/kyr sedimentation rate) of short eccentricity (c.f. Fig. 3 in ref. 2). The two different interpretation would have huge impact on the actual duration estimates of the JSG Unit, with Wang et al. *(1)* suggesting a total duration of ~820 or ~410 Kyr for the 41 m JSG unit from their drill core study (see Fig. 3 in Wang et al. *(1)*, and discussion related to their Fig. 22), whereas the accumulation rate derived from Wu et al. *(2)* predicts a duration of the same stratigraphic interval of >2 Myr. Our new data indicate that the entire Yixian Formation is only 1.633 ± 0.078 Myr max, which means that the JSG Unit within Yixian Formation should be <1.633 Myr. This would clearly exclude the possibility to interpret the 2 m cycle as 100 Kyr eccentricity cycle. It is entirely possible that the sedimentation rates of lacustrine environments between the studied outcrops and drill cores *(1,2, and this study)* are highly variable, obtaining accurate Milankovitch cycle signals from the terrestrial sediments remain a challenging goal without further high resolution and high precision geochronological constraints.

References

1. Y. Q. Wang, P. E. Olsen, J. G. Sha, X. G. Yao, H. Y. Liao, Y. H. Pan, S. Kinney, X. L. Zhang, X. Rao, Stratigraphy, correlation, depositional environments, and cyclicity of the Early Cretaceous Yixian and ?Jurassic-Cretaceous Tuchengzi formations in the Sihetun area (NE China) based on three continuous cores. *Palaeogeogr Palaeoclimatol Palaeoecol* 464, 110–133 (2016).
2. H. C. Wu, S. H. Zhang, G. Q. Jiang, T. S. Yang, J. H., Guo, H. Y. Li. Astrochronology for the Early Cretaceous Jehol Biota in northeastern China. *Palaeogeogr Palaeoclimatol Palaeoecol* 385, 221–228 (2013).

Table S1. Summary of literature age dates from the Yixian Formation in western Liaoning.

Unit / Sample name	Lithology	Method	Mineral	Age (Ma ± 2σ)	Recalculated age* (Ma ± 2σ) [inc. decay constants]	References
**Jiufotang Formation**						
L3001	tuff	SHRIMP U-Pb	zircon	124 ± 4	124 ± 4	*(1)*
Lx9	tuff	$^{40}Ar/^{39}Ar$	K-feldspar	120.1 ± 0.4	121.2 ± 0.7	*(1)*
JFT07-1	tuff	$^{40}Ar/^{39}Ar$	sanidine	122.1 ± 0.3	123.4 ± 0.6	*(2)*
**Yixian Formation**						
**Huanghuashan Unit**						
YX291	rhyolite	LA-ICP-MS	zircon	119.8 ± 3.8	119.8 ± 3.8	*(3)*
YX275	rhyolite	LA-ICP-MS	zircon	118.9 ± 2.8	118.9 ± 2.8	*(3)*
6	volcanic breccia	$^{40}Ar/^{39}Ar$	plagioclase	121.5 ± 1.8	121.7 ± 2.1	*(4)*
6	volcanic breccia	$^{40}Ar/^{39}Ar$	biotite	121.6 ± 1.0	121.8 ± 1.3	*(4)*
**Upper Lava Unit**						
ZCZ28	andesite	$^{40}Ar/^{39}Ar$	groundmass	122.0 ± 1.3	122.9 ± 1.1	*(5)*
Mt14	andesite	$^{40}Ar/^{39}Ar$	groundmass	120.2 ± 1.5	120.2 ± 1.5	*(5)*
Mt9	andesite	$^{40}Ar/^{39}Ar$	groundmass	121.2 ± 1.3	121.2 ± 1.3	*(5)*
	basalt	$^{40}Ar/^{39}Ar$	groundmass	123.7 ± 0.9	123.7 ± 0.9	*(6)*
**Jianshangou Unit**						
YX07-3	tuff	$^{40}Ar/^{39}Ar$	sanidine	122.9 ± 0.7	124 ± 0.9	*(2)*
99L-HDZ1	tuff	$^{40}Ar/^{39}Ar$	sanidine	125.0 ± 0.38	126.2 ± 0.8	*(7)*

Unit / Sample name	Lithology	Method	Mineral	Age (Ma ± 2σ)	Recalculated age* (Ma ± 2σ) [inc. decay constants]	References
LX-HBJ-6	tuff	SHRIMP U-Pb	zircon	122.8 ± 1.6	122.8 ± 1.6	*(8)*
YX07-4	tuff	$^{40}Ar/^{39}Ar$	sanidine	124.1 ± 0.4	125.3 ± 0.7	*(2)*
P1T-2	tuff	TIMS U-Pb	zircon	125.65 ± 0.17	125.65 ± 0.31	*(9)*
P1T-2	tuff	$^{40}Ar/^{39}Ar$	sanidine	124.6 ± 0.6	125.8 ± 0.7	*(10)*
P4T-1	tuff	$^{40}Ar/^{39}Ar$	sanidine	124.6 ± 0.4	125.8 ± 0.8	*(10)*
LX-SHT-12	tuff	SHRIMP U-Pb	zircon	124.7 ± 2.7	124.7 ± 2.7	*(8)*
99L-S1	tuff	$^{40}Ar/^{39}Ar$	sanidine	125.0 ± 0.36	126.2 ± 0.8	*(7)*
YL31	tuff	TIMS U-Pb	zircon	125.2 ± 0.9	125.2 ± 0.9	*(11)*
**Lower Lava Unit**						
	basalt	$^{40}Ar/^{39}Ar$	groundmass	125.0 ± 1.4	125 ± 1.4	*(6)*
YX07-2	basalt	$^{40}Ar/^{39}Ar$	groundmass	126.2 ± 0.4	127.3 ± 0.8	*(2)*
YX07-6	basalt	$^{40}Ar/^{39}Ar$	groundmass	129.7 ± 0.5	130.9 ± 0.8	*(2)*
YL29	basaltic andesite	$^{40}Ar/^{39}Ar$	whole rock	128.2 ± 1.6	131.3 ± 1.8	*(12)*
SYNU-005	basalt	$^{40}Ar/^{39}Ar$	groundmass	126.0 ± 0.8	126 ± 1.1	*(13)*
SYNU-010	basalt	$^{40}Ar/^{39}Ar$	groundmass	125.8 ± 1.0	125.8 ± 1	*(13)*
**Lujiatun Unit**						
L3004	tuff	$^{40}Ar/^{39}Ar$	K-feldspar	123.3 ± 1.3	124.2 ± 1.8	*(14)*
L3003	tuff	$^{40}Ar/^{39}Ar$	K-feldspar	123.0 ± 1.4	124.5 ± 1.9	*(14)*
LX-HBJ-1	tuff	SHRIMP U-Pb	zircon	124.9 ± 1.7	124.9 ± 1.7	*(8)*

* Recalculated age is after Olierook et al *(15)*.

Table S2. U-Pb isotopic data of analyzed single zircon grains by CA-ID-IRMS.

	Compositional Parameters				Radiogenic Isotope Ratios								Isotopic Ages					
Sample / Analysis ID	Th/ U	Pb^*/ Pb_c	Pb_c (pg)	$^{206}Pb/^{204}Pb$	$^{208}Pb/^{206}Pb$	$^{207}Pb/^{206}Pb$	% err	$^{207}Pb/^{235}U$	% err	$^{206}Pb/^{238}U$	% err	correlation coefficient	$^{207}Pb/^{206}Pb$	±	$^{207}Pb/^{235}U$	±	$^{206}Pb/^{238}U$	±
	(a)	(b)	(b)	(c)	(d)	(d)	(e)	(d)	(e)	(d)	(e)		(f)	(e)	(f)	(e)	(f)	(e)
**CY17-9**																		
CY1_6	0.47	11.99	1.0	765.8	0.1485	0.0484	0.87	0.1314	0.93	0.0197	0.08	0.78	116	21	125.31	1.10	125.80	0.10
CY1_7	0.46	12.66	0.9	808.2	0.1472	0.0486	0.91	0.1319	0.97	0.0197	0.10	0.70	127	21	125.77	1.15	125.71	0.12
CY1_8	0.73	16.47	0.9	977.5	0.2322	0.0487	1.11	0.1322	1.19	0.0197	0.09	0.83	132	26	126.06	1.41	125.77	0.11
CY1_9	0.70	2.57	4.0	168.7	0.2231	0.0484	2.94	0.1312	3.11	0.0197	0.23	0.78	118	69	125.17	3.66	125.56	0.28
**CY17-18**																		
CY1_1	0.79	17.55	1.6	1018.1	0.2538	0.0489	0.52	0.1327	0.56	0.0197	0.06	0.66	141	12	126.49	0.67	125.71	0.08
CY1_2	0.63	7.53	1.1	464.8	0.2041	0.0492	1.41	0.1334	1.51	0.0197	0.10	0.99	156	33	127.13	1.80	125.59	0.13
CY1_3	1.01	4.06	1.5	238.1	0.3227	0.0487	3.86	0.1323	4.10	0.0197	0.29	0.85	134	91	126.16	4.87	125.75	0.36
CY4_10	0.60	4.88	2.7	311.6	0.1889	0.0481	1.68	0.1306	1.81	0.0197	0.17	0.80	105	40	124.66	2.12	125.70	0.21
**CY17-17**																		
CY3_6	1.04	10.36	1.4	579.0	0.3298	0.0482	0.98	0.1307	1.05	0.0197	0.09	0.77	110	23	124.69	1.23	125.45	0.11
CY3_7	1.08	4.92	3.5	282.8	0.3409	0.0482	2.43	0.1306	2.57	0.0197	0.16	0.89	108	57	124.64	3.02	125.53	0.20
CY3_8	1.12	9.30	1.4	512.9	0.3531	0.0481	1.15	0.1304	1.22	0.0197	0.09	0.83	104	27	124.44	1.43	125.51	0.11
CY3_9	1.05	10.68	1.3	593.2	0.3356	0.0488	1.06	0.1322	1.13	0.0197	0.08	0.85	138	25	126.08	1.34	125.45	0.10
CY3_10	1.48	10.70	1.1	542.7	0.4725	0.0486	1.20	0.1316	1.28	0.0196	0.09	0.94	128	28	125.52	1.51	125.40	0.11
**CY18-20**																		
CY4_2	1.2	57.9	0.9	3037.5	0.3726	0.0485	0.26	0.1299	0.28	0.0194	0.05	0.53	122	6	124.03	0.33	124.11	0.06
CY4_6	1.1	10.1	4.0	554.1	0.3501	0.0485	1.02	0.1299	1.10	0.0194	0.09	0.85	121	24	124.00	1.28	124.16	0.11
CY4_7	1.1	3.6	2.5	208.6	0.3596	0.0477	2.64	0.1278	2.84	0.0194	0.23	0.89	82	63	122.08	3.27	124.15	0.28
CY4_8	1.0	6.8	2.3	391.3	0.3141	0.0485	1.38	0.1300	1.49	0.0194	0.13	0.85	124	32	124.08	1.74	124.10	0.16
CY4_9	1.0	7.1	3.2	400.9	0.3304	0.0486	1.68	0.1301	1.79	0.0194	0.18	0.69	126	39	124.23	2.10	124.13	0.22

(a) Model Th/U ratio calculated from radiogenic $^{208}Pb/^{206}Pb$ ratio and $^{207}Pb/^{235}U$ age;

(b) Pb^* and Pb_c represent radiogenic and common Pb, respectively;

(c) Measured ratio corrected for spike and fractionation only;

(d) Corrected for fractionation, spike, and common Pb; all common Pb was assumed to be blank with a composition of $^{206}Pb/^{204}Pb = 16.96 \pm 0.35$; $^{207}Pb/^{204}Pb = 14.48 \pm 0.29$; $^{208}Pb/^{204}Pb = 35.03 \pm 0.7$;

(e) Errors are 2-sigma, propagated using the algorithms of Schmitz and Schoene *(16)* and Crowley et al. *(17)*;

(f) Calculations are based on the decay constants of Jaffey et al. *(18)* and a $^{238}U/^{235}U$ ratio of 137.818 (Hiess et al. *(19)*).

Table S3. Mineral compositions (%) of the volcanic tuff samples from Lujiatun and Jianshangou units.

Sample	Illite	Smectite	I-S*	Quartz	Feldspar	Calcite	Zeolite
**Lujiatun Unit**							
CY17-9	4.4		20.8	8.8	31.6	34.4	
CY17-10	8.2		26.7	25.6	37.8		1.6
CY17-18		62		11.1	26.9		
**Jianshangou Unit**							
CY17-16		94.9		0.8	4.3		
CY17-17		91.5		1.3	7.2		
CY17-23		87.7		1	11.2		
CY17-24		90.8			3.1	6.1	

I-S*: Intermediate product between illite and smecitite

Table S4. Major and trace elements compositions (%) of the volcanic tuff samples from Lujiatun, Jianshangou and Huanghuashan units.

Sample	CY17-9	CY17-10	CY17-18	CY17-16	CY17-23	CY17-24	CY17-17	CY18-20
	**Lujiatun Unit**			**Jianshangou Unit**				**Huanghuashan Unit**
Major elements								
SiO_2	42.32	66.98	63.88	57.92	60.37	48.42	60.09	64.53
TiO_2	0.17	0.50	0.64	0.24	0.23	0.13	0.23	0.50
Al_2O_3	10.00	14.59	15.16	16.35	16.06	13.38	17.05	18.27
Fe_2O_3	1.68	3.42	4.22	7.61	3.71	5.48	4.37	2.75
MnO	0.41	0.05	0.03	0.05	0.02	0.13	0.04	0.03
MgO	1.05	1.70	3.79	5.05	5.56	4.18	4.40	0.60
CaO	21.45	2.76	2.75	2.81	2.88	12.21	3.03	1.74
Na_2O	1.93	2.95	2.03	1.11	1.15	1.08	1.92	6.31
K_2O	2.80	3.61	2.11	1.94	2.09	1.82	2.47	4.19
P_2O_5	0.11	0.17	0.17	0.08	0.05	0.05	0.05	0.18
L.O.I	17.49	2.94	4.91	7.10	7.41	12.92	6.40	0.70
Total	99.43	99.67	99.67	100.25	99.52	99.79	100.04	99.81
Trace elements								
Li	24.5	34.9	60.4	124	117	76.5	95.0	12.4
Be	1.55	2.24	2.27	1.70	2.05	1.49	1.89	2.23
Sc	3.16	6.86	9.07	4.95	4.77	3.49	3.88	4.94
V	19.9	48.0	68.5	19.2	18.0	16.7	18.2	40.9
Cr	12.3	30.7	54.5	37.6	5.28	1.88	0.81	85.0
Co	4.74	6.80	15.3	5.47	4.48	8.45	4.01	5.61
Ni	11.3	13.6	28.0	41.5	15.3	35.1	14.8	15.3
Cu	5.32	10.4	16.5	13.1	3.81	6.30	4.13	11.7
Zn	27.0	57.4	61.7	92.8	45.4	52.1	46.9	46.9
Ga	13.1	18.7	19.5	21.2	21.5	14.7	21.6	17.2
Rb	76.3	125	88.2	47.2	53.4	33.7	45.9	137
Sr	291	445	541	283	185	157	293	478
Y	12.5	23.1	18.3	8.46	8.07	11.4	14.6	9.94

Sample	CY17-9	CY17-10	CY17-18	CY17-16	CY17-23	CY17-24	CY17-17	CY18-20
	**Lujiatun Unit**			**Jianshangou Unit**				**Huanghuashan Unit**
Zr	112	233	179	157	225	127	224	192
Nb	12.2	14.2	12.5	13.4	16.2	9.42	13.2	14.7
Sn	1.14	1.93	1.64	2.45	2.70	2.02	2.67	36.0
Cs	1.78	5.36	3.87	0.85	1.23	1.40	0.77	0.86
Ba	383	629	581	682	371	225	804	1284
La	27.4	38.3	37.0	56.4	30.3	34.0	56.5	41.9
Ce	46.1	77.3	70.2	108	61.0	64.3	112	76.9
Pr	5.07	8.77	8.00	10.7	6.43	6.29	11.9	7.81
Nd	17.8	32.2	28.6	34.1	22.3	21.1	41.9	26.2
Sm	3.21	6.05	5.09	5.07	3.84	3.34	7.12	4.04
Eu	0.66	1.22	1.23	0.87	0.65	0.50	1.22	0.94
Gd	2.58	4.76	4.04	3.17	2.57	2.48	4.74	2.48
Tb	0.39	0.70	0.59	0.43	0.36	0.37	0.67	0.35
Dy	2.17	4.07	3.23	2.09	1.90	2.18	3.39	1.88
Ho	0.41	0.80	0.62	0.36	0.33	0.40	0.61	0.37
Er	1.20	2.31	1.77	1.05	0.92	1.16	1.56	1.01
Tm	0.16	0.33	0.24	0.13	0.12	0.16	0.21	0.15
Yb	1.10	2.15	1.63	0.88	0.77	1.02	1.24	0.94
Lu	0.16	0.32	0.23	0.13	0.11	0.16	0.16	0.14
Hf	2.78	6.07	4.71	4.61	6.20	3.95	6.16	4.72
Ta	0.91	1.04	0.89	1.12	1.25	0.94	1.24	0.93
Tl	0.48	0.69	0.87	0.45	0.61	1.16	0.85	0.57
Pb	18.6	22.3	18.7	46.7	26.5	41.8	38.8	20.9
Th	6.34	10.5	9.16	17.1	16.0	15.2	16.0	9.39
U	1.54	2.19	4.26	4.31	2.41	2.87	3.71	1.49
∑REE	108.51	179.32	162.48	222.99	131.58	137.46	243.69	165.11
δEu	0.68	0.67	0.80	0.62	0.59	0.51	0.61	0.84
$(La/Yb)_{CN}$	17.88	12.80	16.31	45.76	28.21	23.91	32.56	31.98

References

1. H. Y. He, X. L. Wang, Z. H. Zhou, F. Wang, A. Boven, G. H. Shi, R. X. Zhu, Timing of the Jiufotang Formation (Jehol Group) in Liaoning, northeastern China, and its implications. *Geophy. Res. Lett.* 31, L12605 (2004).
2. S. C. Chang, H. Zhang, P. R. Renne, Y. Fang, High-precision $^{40}Ar/^{39}Ar$ age for the Jehol Biota. *Palaeogeogr. Palaeoclimatol. Palaeoecol.* 280, 94–104 (2009).
3. H. Zhang, X. M. Liu, W. Chen, Z. T. Li, F. L. Yang, The age of the top of the Yixian Formation in the Beipiao-Yixian area, western Liaoning, and its importance (in Chinese with English abstract). Geol. China 32, 596–603 (2005).
4. P. E. Smith, N. M. Evensen, D. York, M. M. Chang, F. Jin, J. L. Li, S. Cumbaa, D. Russell, Dates and rates in ancient lakes: ^{40}Ar-^{39}Ar evidence for an Early Cretaceous age for the Jehol Group, northeast China. *Can. J. Earth Sci.* 32, 1426–1431 (1995).
5. H. Y. He, Y. X. Pan, L. Tauxe, H. F. Qin, R. X. Zhu, Toward age determination of the M0r (Barremian-Aptian boundary) of the Early Cretaceous. *Phys. Earth Planet. Interi.* 169, 41–48 (2008).
6. R. X. Zhu, Y. X. Pan, R. Shi, Q. Liu, D. Li, Palaeomagnetic and $^{40}Ar/^{39}Ar$ dating constraints on the age of the Jehol Biota and the duration of deposition of the Sihetun fossil-bearing lake sediments, northeast China. *Cret. Res.* 28, 171–176 (2007).
7. C. C. Swisher, X. Wang, Z. H. Zhou, Y. Wang, F. Jin, J. Zhang, X. Xu, F. Zhang, Y. Wang, Further support for a Cretaceous age for the feathered-dinosaur beds of Liaoning, China: New $^{40}Ar/^{39}Ar$ dating of the Yixian and Tuchengzi Formations. *Chinese Sci. Bull.* 47, 136–139 (2002).
8. W. Yang, S. G. Li, B. Y. Jiang, New evidence for Cretaceous age of the feathered dinosaurs of Liaoning: zircon U-Pb SHRIMP dating of the Yixian Formation in Sihetun, Northeast China. *Cret. Res.* 28, 177–182 (2007).
9. P. R. Renne, R. Mundil, G. Balco, K. Min, K. R. Ludwig, Joint determination of ^{40}K decay constants and $^{40}K^{*}/^{40}K$ for the Fish Canyon sanidine standard, and improved accuracy for $^{40}K/^{39}K$ geochronology. *Geochim. Cosmochim. Ac.* 74, 5349–5367 (2010).
10. C. C. Swisher, Y. Q. Wang, X. L. Wang, X. Xu, Y. Wang, Cretaceous age for the feathered dinosaurs of Liaoning, China. *Nature* 400, 58–61 (1999).
11. S. S. Wang, Y. Q. Wang, H. G. Hu, H. M. Li, The existing time of Sihetun vertebrate in western Liaoning. *Chinese Sci. Bull.* 46, 779–782 (2001).
12. S. S. Wang, H. G. Hu, P. X. Li, Y. Q. Wang, Further discussion on the geologic age of Sihetun vertebrate assemblage in western Liaoning, China: evidence from Ar-Ar dating (in Chinese with English abstract). *Acta Petrol. Sin.* 4, 663–668 (2001).

13. S .C. Chang, K. Q. Gao, C. F. Zhou, F. Jourdan, New chronostratigraphic constraints on the Yixian Formation with implications for the Jehol Biota. *Palaeogeogr. Palaeoclimatol. Palaeoecol.* 487, 399–406 (2017).
14. H. Y. He, X. L. Wang, Z. H. Zhou, F. Jin, F. Wang, L. K. Yang, X. Ding, A. Boven, R. X. Zhu, $^{40}Ar/^{39}Ar$ dating of Lujiatun Bed (Jehol Group) in Liaoning, northeastern China. *Geophy. Res. Lett.* 33, L04303 (2006).
15. H. K. Olierook, F. Jourdan, R. Merle, Age of the Barremian–Aptian boundary and onset of the Cretaceous Normal Superchron. *Earth-Sci. Rev.* 197 (2019).
16. M. D. Schmitz, B. Schoene, Derivation of isotope ratios, errors, and error correlations for U-Pb geochronology using ^{205}Pb-^{235}U-(^{233}U)-spiked isotope dilution thermal ionization mass spectrometric data. *Geochem. Geophy. Geosy.* 8, Q08006 (2007).
17. J. L. Crowley, B. Schoene, S. A. Bowring, U-Pb dating of zircon in the Bishop tuff at the millennial scale. *Geology* 35, 1123–1126 (2007).
18. H. Jaffey, K. F. Flynn, L. Glendeni, W. C. Bentley, A. M. Essling, Precision Measurement of Half-Lives and Specific Activities of ^{235}U and ^{238}U. *Phys. Rev. C* 4, 1889–1906 (1971).
19. J. Hiess, D. J. Condon, N. McLean, S. R. Noble, $^{238}U/^{235}U$ Systematics in Terrestrial Uranium-Bearing Minerals. *Science* 335, 1610–1614 (2012).
